# Protection against Mitochondrial and Metal Toxicity Depends on Functional Lipid Binding Sites in ATP13A2

**DOI:** 10.1155/2016/9531917

**Published:** 2016-03-17

**Authors:** Shaun Martin, Sarah van Veen, Tine Holemans, Seyma Demirsoy, Chris van den Haute, Veerle Baekelandt, Patrizia Agostinis, Jan Eggermont, Peter Vangheluwe

**Affiliations:** ^1^Laboratory of Cellular Transport Systems, Department of Cellular and Molecular Medicine, KU Leuven, Campus Gasthuisberg O&N 1, Herestraat 49, P.O. Box 802, 3000 Leuven, Belgium; ^2^Laboratory of Cell Death Research and Therapy, Department of Cellular and Molecular Medicine, KU Leuven, Campus Gasthuisberg O&N 1, Herestraat 49, P.O. Box 802, 3000 Leuven, Belgium; ^3^Research Group for Neurobiology and Gene Therapy, Department of Neurosciences, KU Leuven, Kapucijnenvoer 33, Blok i, P.O. Box 7001, 3000 Leuven, Belgium; ^4^Leuven Viral Vector Core, KU Leuven, Kapucijnenvoer 33, Blok i, P.O. Box 7001, 3000 Leuven, Belgium

## Abstract

The late endo-/lysosomal P-type ATPase ATP13A2 (PARK9) is implicated in Parkinson's disease (PD) and Kufor-Rakeb syndrome, early-onset atypical Parkinsonism. ATP13A2 interacts at the N-terminus with the signaling lipids phosphatidic acid (PA) and phosphatidylinositol (3,5) bisphosphate (PI(3,5)P2), which modulate ATP13A2 activity under cellular stress conditions. Here, we analyzed stable human SHSY5Y cell lines overexpressing wild-type (WT) or ATP13A2 mutants in which three N-terminal lipid binding sites (LBS1–3) were mutated. We explored the regulatory role of LBS1–3 in the cellular protection by ATP13A2 against mitochondrial stress induced by rotenone and found that the LBS2-3 mutants displayed an abrogated protective effect. Moreover, in contrast to WT, the LBS2 and LBS3 mutants responded poorly to pharmacological inhibition of, respectively, PI(3,5)P2 and PA formation. We further demonstrate that PA and PI(3,5)P2 are also required for the ATP13A2-mediated protection against the toxic metals Mn^2+^, Zn^2+^, and Fe^3+^, suggesting a general lipid-dependent activation mechanism of ATP13A2 in various PD-related stress conditions. Our results indicate that the ATP13A2-mediated protection requires binding of PI(3,5)P2 to LBS2 and PA to LBS3. Thus, targeting the N-terminal lipid binding sites of ATP13A2 might offer a therapeutic approach to reduce cellular toxicity of various PD insults including mitochondrial stress.

## 1. Introduction

Mitochondria are organelles with a pivotal role in ATP production, intracellular Ca^2+^ signaling, the generation of reactive oxygen species (ROS), and apoptotic cell death [1–3]. Because of high energy demands at locations distant from the cell body, neurons in particular critically depend on healthy and dynamic mitochondria to fuel membrane excitability and to execute neurotransmission and plasticity [4, 5]. Not surprisingly, defective mitochondrial dynamics is implicated in various neurological disorders, including Parkinson's disease (PD), a common progressive movement disorder characterized by a severe loss of dopaminergic neurons in the* substantia nigra pars compacta* [6]. PD is hallmarked by the accumulation of aggregated *α*-synuclein into Lewy bodies in neurons of the* substantia nigra* and specific brain stem, spinal cord, and cortical regions [7, 8], but also mitochondrial defects are common [9–11]. Strong support for mitochondrial dysfunction in PD comes from the observations that 1-methyl-4-phenyl-1,2,3,4-tetrahydropyridine (MPTP), a potent mitochondrial complex I inhibitor, triggers a PD-like syndrome [12, 13]. Moreover, several PD-associated genes, mainly parkin and PINK1, play a role in mitochondrial dynamics and clearance via mitophagy, which strengthens the concept that mitochondrial dysfunction and/or impaired mitochondrial clearance are tightly linked to PD onset [9, 10].

In the present study, we focus on* ATP13A2/PARK9*, encoding a late endo-/lysosomal membrane protein, which belongs to a poorly characterized subfamily of P-type ATPases, namely, the P5-type transporters with unassigned function and substrate specificity. So far, the three-dimensional structure of ATP13A2 is unknown but can be modelled based on the known structures of other P-type ATPases (i.e., SERCA1a, H^+^-ATPase, Na^+^/K^+^-ATPase, and Cu^2+^-ATPase). Mutations in* ATP13A2* are associated with PD and Kufor-Rakeb syndrome, which is a severe early-onset autosomal recessive form of PD with dementia [15]. ATP13A2 provides cellular protection against metal toxicity induced by Mn^2+^ [16, 17], Zn^2+^ [18], and Fe^3+^ [19], which are considered as environmental risk factors of PD. In addition, ATP13A2 provides protection in several models of *α*-synuclein toxicity [16, 18]. Although loss of ATP13A2 leads to lysosomal dysfunction [20], interestingly, a strong link between ATP13A2 and mitochondrial dysfunction/clearance is emerging. Fibroblasts of patients with nonfunctional ATP13A2 exhibit general mitochondrial dysfunction, including decreased ATP production, enhanced oxygen consumption rates, and fragmentation of the mitochondrial network [21]. Furthermore, knockdown (KD) of ATP13A2 in mouse cortical neurons or human neuroblastoma SHSY5Y cells triggers mitochondrial fragmentation and ROS production [22], whereas ATP13A2 deficiency in patient-derived olfactory neurosphere cultures results in Zn^2+^ dyshomeostasis, which contributes to mitochondrial dysfunction [18]. Finally, in a cellular PD model in which SHSY5Y cells were exposed to the mitochondrial complex I inhibitor rotenone to induce mitochondrial stress, ATP13A2 activity confers cytoprotection, since overexpression of WT ATP13A2, but not a catalytic dead mutant, protects against, whereas KD of ATP13A2 exacerbates cell toxicity [14, 23].

**Figure 1:**
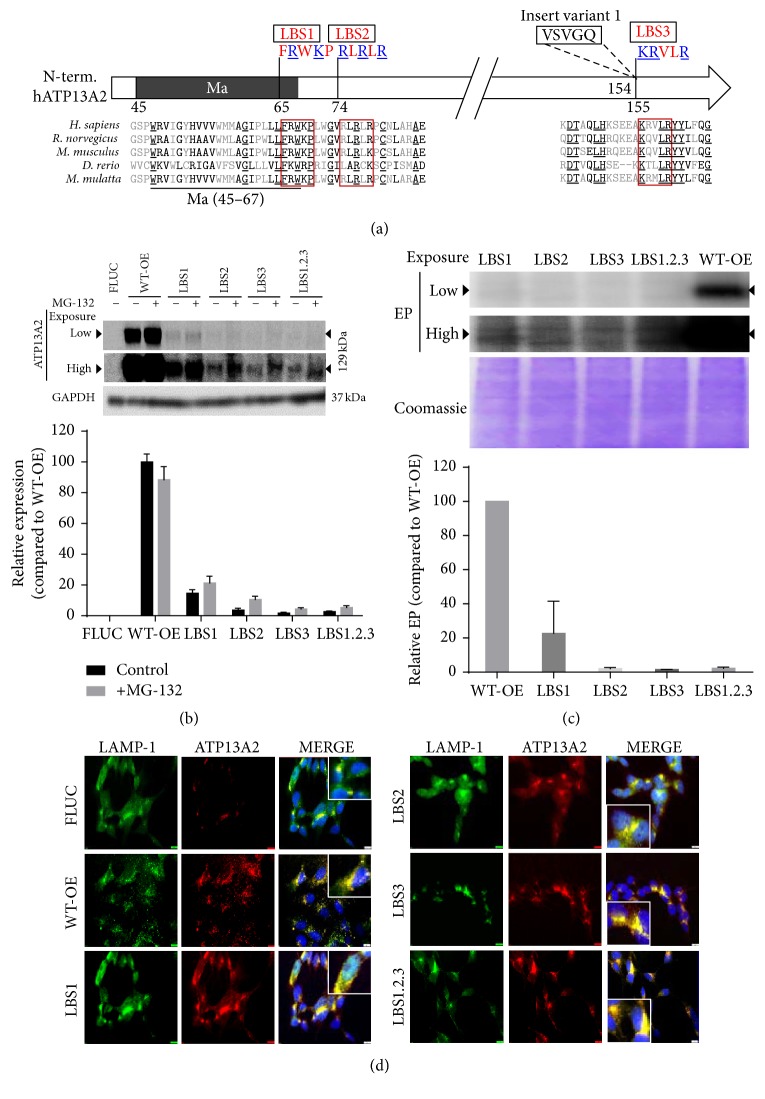
Mutations in the key lipid binding sites of ATP13A2 inhibit activity but not subcellular targeting. (a) Multiple positively charged residues in previously identified lipid binding sites [14] (blue, underlined) were substituted for Ala. LBS3 overlaps with an alternative splicing site rendering an insertion of five additional residues in splice variant 1 of ATP13A2. Ma, membrane-associated region. (b) Expression levels of ATP13A2 were analyzed by immunoblotting with a primary anti-ATP13A2 antibody in comparison to GAPDH as a loading control. Breakdown of LBS mutant proteins by the proteasome was assessed via treatment with the proteasome inhibitor MG-132 (100 *μ*M) for 6 h. The same gel for the expression of ATP13A2 between cell lines has been provided at both low and high intensities. OE, overexpression. (c) Autophosphorylation assay (EP) on microsomes of ATP13A2 WT or LBS mutant expressing SHSY5Y cells. At the top, the same gel was depicted twice at different exposure times. Below, equal protein loading on the SDS-PAGE gel was confirmed by Coomassie staining. We consider that the double bands visible in LBS2 and LBS3 at longer exposure time are background levels while the actual ATP13A2 related EP band (visible in LBS1 and WT-OE) is located in between, but closer to the double band. Quantification of ATP13A2 expression ((b) ATP13A2/GAPDH) and autophosphorylation levels ((c) EP/Coomassie) are depicted. (d) Expression and localization of endogenous (FLUC), WT-OE, LBS1, LBS2, LBS3, or LBS1.2.3 ATP13A2 were confirmed by colocalization experiments with the lysosomal marker LAMP-1 and captured using Olympus IX73 fluorescent microscope. Representative enlarged images of colocalization for all cell lines have been provided as image inserts. Data are representative of 3 independent experiments. Scale bars represent 10 *μ*M.

Of interest, the signaling lipids phosphatidic acid (PA) and phosphatidylinositol (3,5) bisphosphate (PI(3,5)P2) interact at the ATP13A2 N-terminus and stimulate the autophosphorylation reaction, which is a hallmark of its catalytic activity [14]. PA is a conical phospholipid that can alter membrane curvature or act as a local signaling lipid, for example, produced by phospholipase D (PLD). The phosphoinositide PI(3,5)P2 is formed by the PIKfyve lipid kinase and mainly resides in the late endo-/lysosomes where it functions as an organelle tag. Pharmacological inhibition of PLD (to prevent PA production) or PIKfyve (to prevent PI(3,5)P2 generation) counteracts the ATP13A2-mediated protective effect on rotenone-induced mitochondrial toxicity [14, 23]. Together, these results indicate that PA and PI(3,5)P2 are required for the activation of ATP13A2 in conditions of mitochondrial stress. Three putative lipid binding sites (LBS1–3) were previously identified in the ATP13A2 N-terminus via protein lipid binding assays with purified mutant and WT N-terminal protein fragments of ATP13A2 (Figure 1(a)) [14].

To test whether PA and PI(3,5)P2 are key mediators of the cytoprotective effect of ATP13A2 under conditions of mitochondrial stress by direct interaction with and activation of the full length ATP13A2, we here compared SHSY5Y cell lines stably expressing WT ATP13A2 or LBS1–3 mutants. We further explored whether a similar PA- and PI(3,5)P2-dependent ATP13A2 activation mechanism may also provide cellular protection against metal (Mn^2+^, Zn^2+^, and Fe^3+^) induced cytotoxicity.

## 2. Materials and Methods

### 2.1. Cell Culture

SHSY5Y neuroblastoma cell lines stably expressing firefly luciferase (FLUC, control), WT ATP13A2, mutants of the three putative N-terminal LBS (LBS1: ^65^FRWKP→FAWAP; LBS2: ^74^RLRLR→ALALA; LBS3: ^155^KRVLR→AAVLA; LBS1.2.3: combination of LBS1–3 mutations), or sh-ATP13A2 (KD [14, 23]) were generated via lentiviral transduction and maintained as described previously [14].

### 2.2. Drug Treatments

Cells were exposed to rotenone (Rot, 1 *μ*M; R8875, Sigma), MPP+ (50 *μ*M, DO48, Sigma), zinc (ZnCl_2_, 150 *μ*M; Z0152, Sigma), manganese (MnCl_2_, 2 *μ*M; 205891000, Acros Organics), and iron (FeCl_3_, 1.5 mM; 157740, Sigma) for 24 hours (h). The final concentration of each agent was chosen from a dose response analysis to obtain a submaximal inhibition of cell viability. Prior to stressor addition, cells were pretreated for 1 h with YM-201636 (PIKfyve inhibitor, PIK, 200 nM; 524611, Millipore) or 5-fluoro-2-indolyl des-chlorohalopemide (PLD inhibitor, FIPI, 100 nM; F5807, Sigma) to inhibit the production of PI(3,5)P2 or PA, respectively. To inhibit the proteasome, cells were incubated with MG-132 (100 *μ*M; M7449, Sigma) for 6 h.

### 2.3. Fluorescence Microscopy

Cells were fixed with 4% paraformaldehyde at 37°C (30 min) and permeabilized with 0.1% Triton X-100 at room temperature (10 min). After blocking in PBS containing 1% BSA and 10% goat serum for 1 h at room temperature, samples were incubated with primary antibodies targeting ATP13A2 or LAMP-1 (Sigma) overnight at 4°C. Thereafter, samples were washed and exposed to Alexa Fluor 488 (green) or Alexa Fluor 647 (red) secondary antibodies. Cells were counterstained with DAPI (1 *μ*g/mL) for 10 min, mounted using Prolong Gold antifade reagent, and cured overnight. Images were acquired with an Olympus IX73 fluorescent microscope using a 63x objective and dimension cellSens software. Scale bars represent 10 *μ*M.

### 2.4. Cell Death

Cell death was determined by propidium iodide (PI) exclusion. Briefly, cells were trypsinized at the indicated time points and incubated with 1 *µ*g/ml PI. PI-positive (dead) cells were quantified via flow cytometry (Attune Cytometer, Life Technologies).

### 2.5. Cell Viability

Cells were seeded at 5000 cells per well of a 96-well plate. Following treatment cells were washed with PBS and incubated with 0.01 mg/mL MUH (4-methylumbelliferyl heptanoate, Sigma) dissolved in PBS for 30 min at 37°C. Fluorescence was measured with a Flex Station plate reader (Molecular Devices) with excitation 355 nm, emission 460 nm, and cutoff value of 455 nm.

### 2.6. Cellular Fractionation

Cells were seeded in 15 cm dishes at a density of 1.5 × 10^6^. After treatment, cells were harvested and resuspended in hypotonic buffer (10 mM Tris-HCl pH 7.5, 0.5 mM MgCl_2_, and SigmaFast protease inhibitor cocktail (Sigma)) and a 10 min incubation period on ice. Cells were homogenized by applying 40 strokes in a Dounce homogenizer and after adding the 1 M buffer solution (0.5 M sucrose, 10 mM Tris-HCl pH 7.3, 40 *μ*M CaCl_2_, 0.23 *μ*M phenylmethylsulfonyl fluoride, and 1 mM dithiothreitol (DTT)) 20 additional strokes were performed. The total cell lysate was subjected to differential centrifugation: the nuclear fraction (1,000 g; 10 min), the mitochondrial/lysosomal fraction (12,000 g; 20 min), and, lastly, the microsomal and cytosolic fractions (200,000 g; 35 min; pellet and supernatant, resp.). Microsomal pellets were resuspended in a 250 mM sucrose solution supplemented with protease inhibitor cocktail. All fractionation steps were carried out at 4°C. After solubilization, samples were aliquoted, flash-frozen in liquid nitrogen, and stored at −80°C. The protein concentration was determined by the Qubit fluorometric method (Life Technologies).

### 2.7. Immunoblotting

10 *μ*g protein of the microsomal fractions was separated on precast NuPAGE 4–12% BisTris gels using MOPS running buffer (Life Sciences), followed by transfer onto polyvinylidene fluoride membranes (Millipore) according to the manufacturer's instructions. After blocking in TBS (50 mM Tris, 150 mM NaCl, pH 7.5) supplemented with 5% nonfat dry milk and 0.1% Tween-20 (Sigma), blots were incubated for 1 h with primary polyclonal anti-ATP13A2 (1/1,000 dilution; A3361, Sigma) and anti-GAPDH antibodies (1/5,000 dilution; G8795, Sigma) and 45 min with horseradish peroxidase conjugated IgG secondary antibodies (1/2,000 dilution; Bioke). Expression was detected using enhanced chemiluminescence substrate (Pierce) and the Bio-Rad ChemiDoc MP imaging system. Quantification was performed with ImageJ software (http://rsbweb.nih.gov/ij/).

### 2.8. Autophosphorylation Assay

40 *μ*g of the microsomal fraction was added to a final volume of 95 *μ*L of reaction buffer (160 mM KCl, 17 mM Hepes, 2 mM MgCl_2_, 1 mM DTT, and 5 mM NaN_3_). The autophosphorylation assay was started by adding [*γ*-^32^P] ATP (2 *μ*Ci) and stopped after 1 min with 400 *μ*L of ice-cold stop solution (20% trichloroacetic acid, 10 mM phosphoric acid). Samples were incubated on ice for 30 min to precipitate protein and centrifuged at 20,000 g for 30 min at 4°C. The pellet was washed twice with 400 *μ*L of ice-cold stop solution and finally dissolved in sample buffer (10% LDS, 10 mM NaH_2_PO_4_, 0.01% SDS, 10 mM 2-mercaptoethanol, and 0.15 mg/mL bromophenol blue). After loading the samples on NuPAGE 4–12% BisTris gels (Life Sciences), electrophoresis was conducted for 1.5 h (40 mA, 170 V) in running buffer containing 0.1% SDS and 170 mM MOPS (pH 6.3). Following fixation in 7.5% acetic acid, the gel was exposed to a PhosphorImager screen (GE Healthcare) and, the next day, radioactivity was visualized in a PhosphorImager scanner (Storm 860, GE Healthcare). Quantification was performed with Image QuanT (Molecular Dynamics) and ImageJ software packages (http://rsbweb.nih.gov/ij/).

### 2.9. Statistical Analysis

Data are presented as the average ± SD of three independent experiments. Statistical analysis was conducted by one-way analysis of variance (ANOVA) with either Dunnett's or Bonferroni post hoc corrections. Consider ^*∗*/$^ *P* < 0.05, ^*∗∗*/$$^ *P* < 0.01, and  ^*∗∗∗*/$$$^ *P* < 0.001.

## 3. Results

### 3.1. LBS2-3 Mutations Prevent ATP13A2 Activation and Cytoprotection

Previously, using purified N-terminal fragments of ATP13A2 in protein lipid overlay assays, we described that LBS1 and LBS2 are mainly required for interaction with PI(3,5)P2 (PA to a lesser extent), whereas LBS3 is essential for PA interaction [14] (Figure 1(a)). Here, we tested whether the protective effect of ATP13A2 in conditions of rotenone-induced mitochondrial toxicity can be explained by a specific and direct interaction of PI(3,5)P2 and PA to the full length ATP13A2 protein. To that end, we generated stable SHSY5Y cell lines with ATP13A2 KD (75.4 ± 8.9% reduction in ATP13A2 mRNA levels as compared to FLUC [14]) or overexpression of FLUC (firefly luciferase, control), ATP13A2 WT, and four mutants in the putative N-terminal lipid binding sites (LBS1, LBS2, LBS3, and LBS1.2.3).

First, we confirmed by immunolocalization that, like WT ATP13A2 overexpression, the LBS1, LBS2, LBS3, and LBS1.2.3 mutants are also expressed in the LAMP-1 positive organelles of the SHSY5Y cells (Figure 1(d)). Note that the expression of LBS mutants rose well above the endogenous ATP13A2 protein levels (FLUC), since the endogenous expression of ATP13A2 in FLUC is only weakly detectable (Figure 1(d)) [14]. These results show that the LBS mutants are expressed in the late endo-/lysosomes to levels well above the endogenous ATP13A2, which was confirmed by immunoblotting (Figure 1(b), compared to ATP13A2 levels in FLUC cell line which fall below the detection limit [14]), and LBS mutants). According to the immunoblot analysis, the protein levels in the LBS mutant cell lines were at least 10-fold lower than WT. This difference in protein expression between WT and LBS mutants was repeatedly observed when several WT and LBS1–3 clones were evaluated using various viral vector dilutions. In addition, the inhibition of the proteasome by MG-132 only partially enhanced the expression levels of the mutated proteins suggesting that protein instability may not be a major issue (Figure 1(b)).

**Figure 2:**
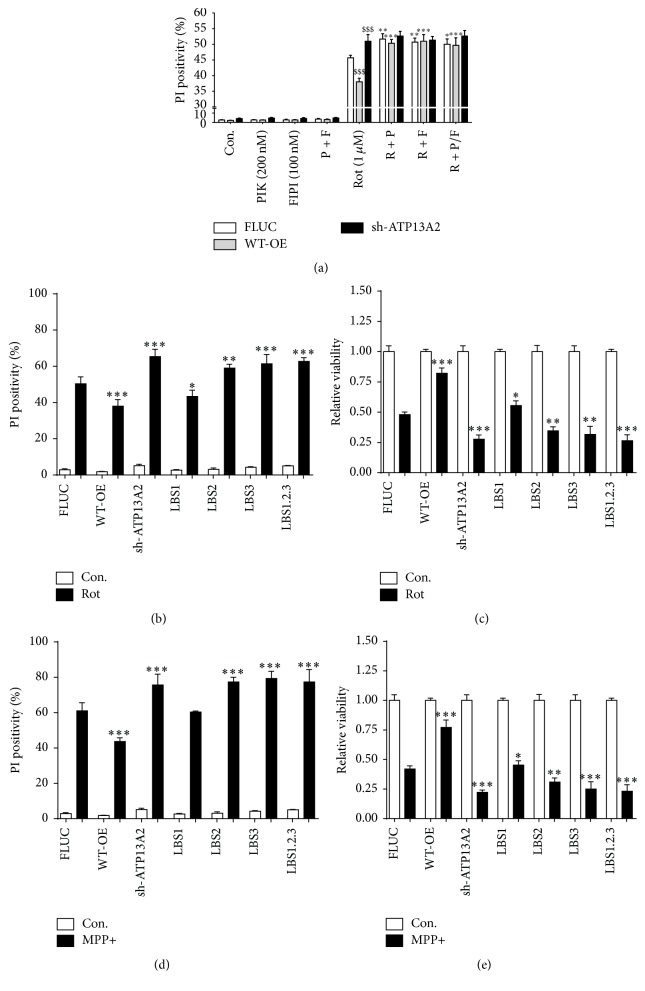
LBS mutations prevent ATP13A2-mediated cytoprotection. (a) ATP13A2's protective response at 24 h exposure to rotenone (Rot, 1 *μ*M) and the effect of pharmacological inhibition of PIKfyve lipid kinase with YM-201636 (PIK or P, 200 nM) and inhibition of PLD with FIPI (F, 100 nM) on cell death were assessed via a propidium iodide (PI) based assay. $ statistical differences between FLUC and WT-OE/sh-ATP13A2, *∗* statistical differences within cell line following treatment with inhibitor (1 mark, *P* < 0.05; 2 marks, *P* < 0.01; 3 marks, *P* < 0.001) (ANOVA with Bonferroni post hoc test). ((b)–(e)) Stable cell lines were exposed to 1 *μ*M rotenone ((b)-(c)) or 50 *μ*M MPP+ ((d)-(e)) for 24 h and cell death was assessed by PI stained flow cytometry, whereas cell viability was assayed by the MUH protocol. Data are the mean of 3 independent experiments ± SD. *∗* statistical differences between FLUC and WT-OE/sh-ATP13A2/LBS1/LBS2/LBS3/LBS1.2.3 (1 mark, *P* < 0.05; 2 marks, *P* < 0.01; 3 marks, *P* < 0.001) (ANOVA with Bonferroni post hoc test).

Next, we confirmed that overexpression of ATP13A2 WT protects, whereas KD of ATP13A2 sensitizes cells to rotenone (Figure 2(a)), in line with our previous findings [14]. Moreover, pharmacological inhibition of the PIKfyve lipid kinase by YM-201636 or of the PLD activity by FIPI prevented the cellular protection in ATP13A2 overexpression cell lines but had no significant effect on the ATP13A2 KD cells, suggesting a direct and activating effect of both lipids on ATP13A2 (Figure 2(a)). Notably, the inhibitors exerted no significant toxic effect in the absence of rotenone (Figure 2(a)).

To further assess the role of ATP13A2 at the level of the mitochondria, we compared the sensitivity of our cell lines to rotenone and MPP+, another complex I inhibitor (Figures 2(b)–2(e)). Data demonstrated that, as for rotenone, ATP13A2 WT protects and KD sensitized cells to MPP+ toxicity (Figures 2(d) and 2(e)). Interestingly, in comparison with WT, overexpression of the LBS2, LBS3, and LBS1.2.3 mutants failed to protect against either rotenone- or MPP+-induced mitochondrial stress, in line with a critical role of the LBS2/3 sites in the ATP13A2-mediated cellular protection (Figures 2(b)–2(e)). Moreover, compared to FLUC, LBS1 demonstrated a slightly weaker than WT, but significant protective effect against rotenone or MPP+ in the cell viability assay. In contrast to rotenone, the protection of LBS1 to MPP+ was not significant in the cell death assay (Figures 2(b) and 2(c)). The failure of the LBS2-3 mutants to protect against either rotenone or MPP+ is not merely related to their lower expression levels, since, for either cell death induction (Figures 2(b) and 2(d)) or inhibition of cell viability (Figures 2(c) and 2(e)) a significant sensitization to either agent was observed in the LBS2, LBS3, and LBS1.2.3 cell lines to the level of ATP13A2 KD, whereas in LBS1 such a sensitization was not observed. Finally, we tested whether mutagenesis of the key lipid interacting sites influenced the autophosphorylation properties of ATP13A2 (Figure 1(c)). No autophosphorylation signal was observed in the LBS2/3/1.2.3 cells, whereas in LBS1 cells a faint, but reproducible autophosphorylation signal was detected.

Altogether, these data pieces point to an inhibitory effect of the LBS2-3 mutations on the functionality of ATP13A2 in conditions of mitochondrial stress.

### 3.2. PA and PI(3,5)P2 Mediated Protection against Mitochondrial Stress Occurs Specifically via ATP13A2

**Figure 3:**
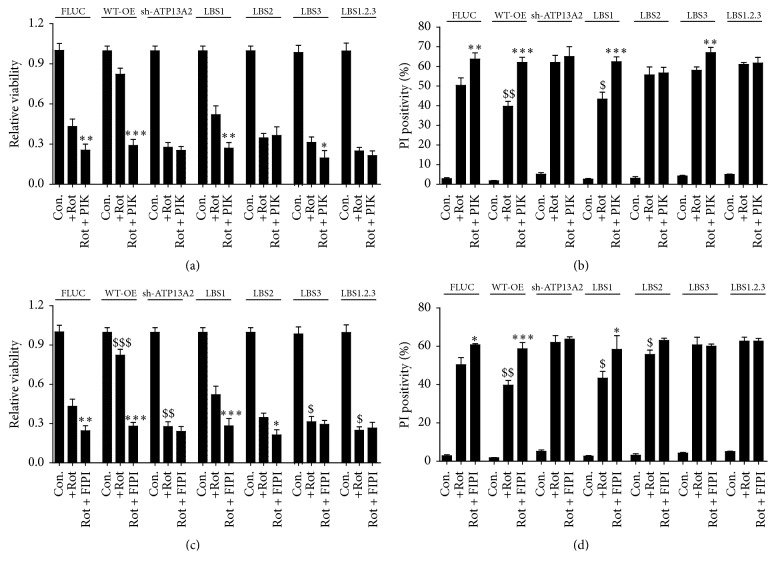
PA and PI(3,5)P2 mediated protection to mitochondrial stress occurs via ATP13A2. SHSY5Y cells stably expressing FLUC, shRNA for KD of ATP13A2, ATP13A2, and full length mutants of ATP13A2 with mutated putative lipid binding sites (LBS1–3) were exposed to rotenone (Rot, 1 *μ*M) in the presence or absence of 1 h pretreatment with the pharmacological inhibitors of PIKfyve lipid kinase (((a) and (b)) YM-201636, 200 nM) or phospholipase D (((c) and (d)) FIPI, 100 nM). Following 24 h exposure to rotenone, cells were assessed for cell viability ((a) and (c)) or death ((b) and (d)) by, respectively, MUH and propidium iodide (PI) assays. Data are the mean of 3 independent experiments ± SD. $ statistical differences between FLUC and WT-OE/sh-ATP13A2/LBS1/LBS2/LBS3/LBS1.2.3, *∗* statistical differences within cell line following treatment with inhibitor (1 mark, *P* < 0.05; 2 marks, *P* < 0.01; 3 marks, *P* < 0.001) (ANOVA with Bonferroni post hoc test).

We investigated whether pharmacological inhibition of PA and PI(3,5)P2 production may influence the response of the LBS cell lines to rotenone-induced mitochondrial stress (Figure 3). In the LBS1 and LBS3 cell lines, inhibition of PIKfyve by YM-201636 significantly increased rotenone-induced toxicity (Figures 3(a) and 3(b)). In contrast, YM-201636 exposure was unable to incite further stress in either the LBS2 or LBS1.2.3 cell lines. In the case of PA, inhibition of PLD by FIPI was unable to potentiate the rotenone-elicited toxicity in LBS3 and LBS1.2.3 (Figures 3(c) and 3(d)). Yet, for the LBS1 cells, FIPI significantly potentiated rotenone-induced toxicity (Figures 3(c) and 3(d)), whereas, in the case of LBS2, a significant effect of FIPI was only observed in the cell viability assay (Figure 3(c)). These observations correlate well with previous observations that PI(3,5)P2 predominantly interacts with LBS2 and PA with LBS3 [14]. Altogether, these results are in line with an inhibitory effect of the N-terminus on ATP13A2 activity, which is reversed by specific and direct binding of PA and PI(3,5)P2 to the LBS sites in conditions of rotenone-induced mitochondrial stress.

### 3.3. PA and PI(3,5)P2 Provide ATP13A2-Mediated Protection to PD-Related Metal Toxicity

**Figure 4:**
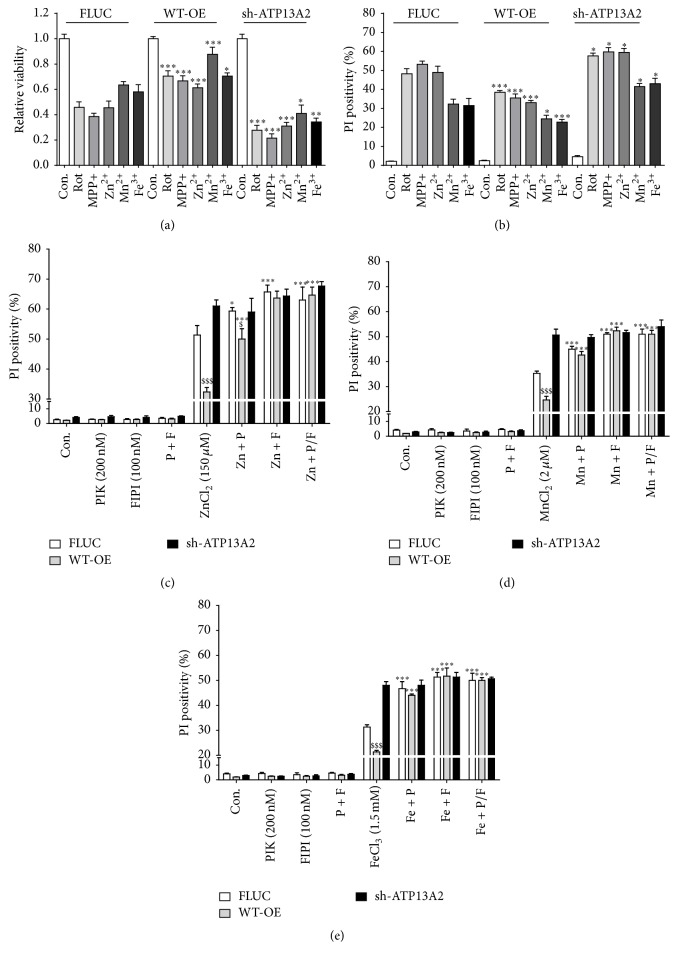
PA and PI(3,5)P2 provide ATP13A2-mediated protection to PD-related metal toxicity. ((a)-(b)) The SHSY5Y cell models stably overexpressing FLUC, WT-OE, or sh-ATP13A2 were exposed for 24 h to rotenone (Rot, 1 *μ*M, positive control) and MPP+ (50 *μ*M), as well as the heavy metals Zn^2+^ (150 *μ*M), Mn^2+^ (2 *μ*M), and Fe^3+^ (1.5 mM). Cell viability was evaluated by the MUH assay (a), whereas cell death was assessed by propidium iodide (PI) stained flow cytometry (b). To test whether the protective role of ATP13A2 depends on the signaling lipids PI(3,5)P2 and PA, the SHSY5Y cell lines were pretreated for 1 h with the PIKfyve inhibitor (200 nM; P or PIK) and/or the phospholipase D inhibitor (100 nM; F or FIPI) prior to the addition of the heavy metals Zn^2+^ (c), Mn^2+^ (d), and Fe^3+^ (e). The level of cellular protection was evaluated by PI stained flow cytometry. Data are the mean of 3 independent experiments ± SD. $ statistical differences between FLUC and WT-OE, *∗* statistical differences within cell line following treatment with inhibitor (1 mark, *P* < 0.05; 2 marks, *P* < 0.01; 3 marks, *P* < 0.001) (ANOVA with Bonferroni post hoc test).

The protective effect of ATP13A2 on various metal ions (Mn^2+^ [16, 17], Zn^2+^ [18], and Fe^3+^ [19]) depends on the catalytic activity of ATP13A2, since a catalytic dead mutant is unable to provide cellular protection. Here, we tested whether the activation of ATP13A2 during metal toxicity also depends on PA and/or PI(3,5)P2. In the stable SHSY5Y cell lines we observed that similar to rotenone and MPP+ toxicity ATP13A2 overexpression protected against, whereas KD sensitized cells to metal toxicity following exposure to Zn^2+^, Mn^2+^, and Fe^3+^ (Figures 4(a) and 4(b)), in line with previous reports [14, 16, 17, 19, 23].

Next, we addressed whether the ATP13A2-mediated protection to metal toxicity also depends on PA and PI(3,5)P2 interaction. Similar to that in conditions of rotenone (Figure 2(a)), inhibition of PI(3,5)P2 formation significantly blunted the protective effect following metal exposure observed in both FLUC and WT overexpression cells, but not in the KD cell line (Figures 4(c)–4(e)). Like the data obtained for YM-201636, FIPI also blunted the protective effect of ATP13A2 on the metal ions Zn^2+^, Mn^2+^, and Fe^3+^ (Figures 4(c)–4(e)). However, unlike YM-201636, FIPI increased the sensitivity of FLUC and WT overexpression cells up to the level of the ATP13A2 KD cells, whereas coexposure of YM-201636 and FIPI did not further potentiate cell death. Finally, in ATP13A2 KD cells YM-201636 or FIPI did not exert an effect on metal ion toxicity, whereas in all cell lines treatment with either YM-201636 or FIPI alone did not induce cell death (Figures 4(c)–4(e)).

**Figure 5:**
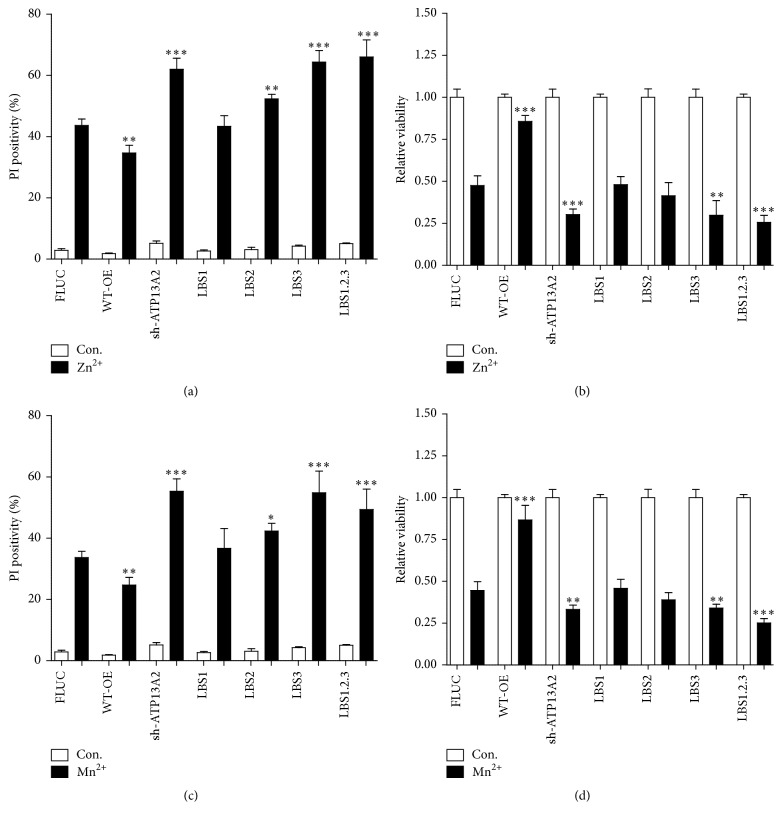
The capacity of ATP13A2 to interact with PA/PI(3,5)P2 is essential to protect against heavy metal-induced cytotoxicity. SHSY5Y cell lines stably overexpressing FLUC, ATP13A2 shRNA, ATP13A2 WT, and LBS1–3 mutants were exposed to toxic heavy metal concentrations of Zn^2+^ (150 *μ*M ((a) and (b))) or Mn^2+^ (2 *μ*M ((c) and (d))). Cell death induction was assessed by propidium iodide (PI ) based flow cytometry ((a) and (c)) and cell viability by MUH assay ((b) and (d)). Data are the mean of 3 independent experiments ± SD. *∗* statistical differences between FLUC and WT-OE/sh-ATP13A2/LBS1/LBS2/LBS3/LBS1.2.3 (1 mark, *P* < 0.05; 2 marks, *P* < 0.01; 3 marks, *P* < 0.001) (ANOVA with Bonferroni post hoc test).

In addition, the LBS1–3 cells were exposed to the metal ions Zn^2+^ or Mn^2+^, and cytotoxicity and cell death were assessed (Figure 5). Compared to ATP13A2 WT and FLUC, LBS3 and LBS1.2.3 showed a significant loss in the protective effect of ATP13A2 towards either Zn^2+^ or Mn^2+^ (Figures 5(a)–5(d)). Although LBS2 demonstrated a significant, albeit small, reduction in the protective capacity based on the cell death assay, no significant difference was found for LBS2 in the cell survival assay, suggesting that the sensitization to heavy metal toxicity was most consistent and significant in the LBS3 cell line.

## 4. Discussion

### 4.1. Inhibition of ATP13A2 Activity by the N-Terminus Is Reversed by PA and PI(3,5)P2 Interaction

The catalytic activity of ATP13A2 provides cellular protection against various PD-related insults like mitochondrial stress [14, 23] and metal exposure (Mn^2+^ [16, 17], Zn^2+^ [18], and Fe^3+^ [19]). Here we established that these various cytoprotective effects depend on the same activation mechanism of ATP13A2 involving an N-terminal lipid switch. Our results show that both PI(3,5)P2 and PA are required to exert ATP13A2-mediated cellular protection, which is explained by a specific and direct interaction of PI(3,5)P2 and PA at the ATP13A2 N-terminal lipid binding sites LBS2 and LBS3, respectively.

Whereas LBS1.2.3 mutants do not respond to either PIKfyve or PLD inhibition, pharmacological inhibition of PIKfyve or PLD had no impact on rotenone toxicity in, respectively, the LBS2 and LBS3 cell lines, strongly indicating that PI(3,5)P2 binds to LBS2 and PA to LBS3. This is in agreement with previous results of protein lipid overlays with purified N-terminal fragments of ATP13A2 [14]. In addition, our data demonstrate that both lipids need to bind together to allow ATP13A2 activation, since pharmacological inhibition of only PA or PI(3,5)P2 formation is sufficient to abolish the cellular protection of ATP13A2 WT to rotenone. No autophosphorylation signal was detected for the LBS2/3/1.2.3 mutants, whereas a weak signal was detected for LBS1, suggesting that the LBS2/3/1.2.3 mutants might exhibit loss of autophosphorylation activity. However, we cannot fully exclude that the autophosphorylation signal might have fallen below the detection level of the assay due to the lower expression levels of the mutants. But since we previously demonstrated that application of PA and PI(3,5)P2 stimulates the autophosphorylation reaction [14] and we now show that the protective effects of ATP13A2 depend on lipid interactions on LBS2/3, it is reasonable to speculate that the functional LBS2-3 sites are required for the catalytic autophosphorylation reaction.

Together, our observations highlight the critical dependence of ATP13A2 activation on the lipid interactions at the membrane-associated N-terminal domain and suggest that the N-terminus might be an autoinhibitory domain preventing ATP13A2 activity. Autoinhibition of P-type ATPases by N- or C-terminal regions is frequently observed, for example, in the human plasma membrane Ca^2+^-ATPase PMCA [24] and the proton pump in plants [25].

Surprisingly, the LBS2/3/1.2.3 but not LBS1 cell lines displayed an increased sensitivity to either rotenone or MPP+ in comparison to FLUC control cells and reached similar toxicity levels as observed for KD cells. This result may point to a dominant negative effect of the LBS2/3/1.2.3 mutants suppressing the protective effect of the endogenous ATP13A2. Of interest, this dominant negative effect was not observed with LBS1 (Figure 2) or the catalytic dead mutant D508N [14], which both contain intact LBS2/3 sites. The LBS2/3 mutated N-terminus might therefore inhibit the endogenous ATP13A2 by direct interaction or by irreversibly trapping regulatory proteins and/or lipids required for ATP13A2 activation. Alternatively, the endogenous WT ATP13A2 levels might be reduced upon expression of the LBS2/3/1.2.3 mutants, which might also explain why the LBS mutant cell lines display an increased sensitivity to mitochondrial stress as compared to control cells. These possibilities will be addressed in future experiments.

### 4.2. Lysosomal and Mitochondrial Dysfunctions Are Tightly Connected in PD

So far the exact cellular function of ATP13A2 remains obscure, since the transported substrate of ATP13A2 is not yet identified. However, a role of ATP13A2 in the clearance of mitochondria and proteins is gradually emerging. ATP13A2 is involved in the biogenesis and release of exosomes mediating *α*-synuclein clearance [18], whereas a role of ATP13A2 in autophagy-dependent pathways has also been proposed, although mechanistic details are lacking [21, 22, 26]. Indeed, ATP13A2 KD in SHSY5Y cells reduces autophagic flux [22], whereas lysosomal mediated clearance of autophagosomes is impaired in patient-derived ATP13A2^−/−^ fibroblasts [20]. A decreased autophagic flux associated with ATP13A2 deficiency may critically affect mitochondrial quality control, which may explain mitochondrial fragmentation and elevated ROS production [22].

Of interest, the dependency of ATP13A2 activation on PA and PI(3,5)P2 further connects ATP13A2 to autophagy-mediated clearance pathways [14, 23]. Indeed, PLD1 regulates *α*-synuclein clearance via autophagy [27], whereas PI(3,5)P2 regulates endo-/lysosome morphology and is also implicated in autophagy [28, 29]. By stimulating ATP13A2 activity both lipids might regulate autophagy and promote mitochondrial quality control and overall cellular health. ATP13A2 activity might be specifically required in conditions of mitochondrial stress and damage, which can be induced by various insults, such as the complex I inhibitors rotenone/MPP+ or also by toxic concentrations of Zn^2+^, Mn^2+^, or Fe^3+^ [30, 31]. Indeed, loss of ATP13A2 in olfactory neurosphere cultures results in Zn^2+^ dyshomeostasis, which on its term contributes to mitochondrial dysfunction [18].

PD studies highlight the importance of mitochondrial maintenance and clearance by PINK1/parkin mediated mitophagy, a macroautophagy pathway involving the encapsulation of defective mitochondria in autophagosomes [32–34]. But how lysosomes accept the mitochondria for subsequent degradation via mitophagy pathways or alternative routes such as mitochondrial derived vesicles [35] and what the role of ATP13A2 is herein remain poorly understood. Nevertheless, lysosomal and mitochondrial dysfunctions may be tightly connected in PD. Besides ATP13A2, also mutations in the lysosomal protein glucocerebrosidase (GBA) are genetic risk factors for PD leading to impaired lysosomal sphingolipid degradation, mitochondrial fragmentation, and elevated ROS levels [36]. In addition, LRRK2 affects lysosomal functionality and regulates mitochondrial dynamics [37, 38].

In conclusion, PA and PI(3,5)P2 are required for ATP13A2-mediated protection to rotenone/MPP+-induced mitochondrial stress and toxic Mn^2+^/Zn^2+^/Fe^3+^ concentrations, suggesting a general lipid-dependent ATP13A2 activation mechanism that relieves the N-terminal autoinhibition. Thus, targeting the N-terminal lipid binding sites of ATP13A2 might offer a therapeutic modality to activate ATP13A2 and reduce cellular toxicity of various PD insults.
